# Isolation and Genetic Analysis of Multidrug Resistant Bacteria from Diabetic Foot Ulcers

**DOI:** 10.3389/fmicb.2015.01464

**Published:** 2016-01-05

**Authors:** Shailesh K. Shahi, Ashok Kumar

**Affiliations:** School of Biotechnology, Faculty of Science, Banaras Hindu UniversityVaranasi, India

**Keywords:** diabetic foot ulcer, antibiotic resistance, PCR, class 1 integron, β-lactamase, multidrug resistance

## Abstract

Severe diabetic foot ulcers (DFUs) patients visiting Sir Sunderlal Hospital, Banaras Hindu University, Varanasi, were selected for this study. Bacteria were isolated from swab and deep tissue of 42 patients, for examining their prevalence and antibiotic sensitivity. DFUs of majority of the patients were found infected with *Enterococcus* spp. (47.61%), *Escherichia coli* (35.71%), *Staphylococcus* spp. (33.33%), *Alcaligenes* spp. (30.95%), *Pseudomonas* spp. (30.95%), and *Stenotrophomonas* spp. (30.95%). Antibiotic susceptibility assay of 142 bacteria with 16 antibiotics belonging to eight classes showed the presence of 38 (26.76%) isolates with multidrug resistance (MDR) phenotypes. MDR character appeared to be governed by integrons as class 1 integrons were detected in 26 (68.42%) isolates. Altogether six different arrays of genes (*aad*A1, *aad*B, *aad*AV, *dhfr*V, *dhfr*XII, and *dhfr*XVII) were found within class 1 integron. Gene cassette *dhfr*AXVII-*aad*AV (1.6 kb) was present in 12 (3 Gram positive and 9 Gram negative) isolates and was conserved across all the isolates as evident from RFLP analysis. In addition to the presence of class 1 integron, six β-lactamase resistance encoding genes namely *bla*_TEM_, *bla*_SHV_, *bla*_OXA_, *bla*_CTX−M−gp1_, *bla*_CTX−M−gp2_, and *bla*_CTX−M−gp9_ and two methicillin resistance genes namely *mecA* and *femA* and vancomycin resistance encoding genes (*vanA* and *vanB*) were identified in different isolates. Majority of the MDR isolates were positive for *bla*_TEM_ (89.47%), *bla*_OXA_ (52.63%), and *bla*_CTX−M−gp1_ (34.21%). To our knowledge, this is the first report of molecular characterization of antibiotic resistance in bacteria isolated from DFUs from North India. In conclusion, findings of this study suggest that class-1 integrons and β-lactamase genes contributed to the MDR in above bacteria.

## Introduction

Diabetic foot infections (DFIs) constitute a major clinical and financial burden to the diabetic patients (Bakker et al., 2005; Singh et al., 2005; Turhan et al., 2015). DFIs are one of the most feared complications of diabetes that can progress rapidly to irreversible septic gangrene necessitating amputation of the foot. Patients with diabetes are 25 times more likely to lose a leg than those without diabetes, and up to 70% of all leg amputations occur in people with diabetes (Singh et al., 2005). The specific organisms found in DFIs differ not only from patient to patient, but also from one part of the country to another (Ozer et al., 2010). Individuals with diabetes have higher rates of hospitalization for soft-tissue and bone infections of the foot than patients without diabetes (Sotto et al., 2012). Microbiological studies have indicated polymicrobial nature of DFIs, the most frequently identified isolates being aerobes including *Staphylococcus aureus, Staphylococcus epidermidis*, coagulase-negative *Staphylococcus* spp., *Enterococcus* spp., *Escherichia coli, Pseudomonas aeruginosa, Proteus mirabilis*, and *Klebsiella pneumoniae* (Turhan et al., 2015). The most common anaerobic isolates are *Peptostreptococcus* spp, *Bacteroides fragilis*, and *Clostridium* spp. (Dowd et al., 2008).

Antibiotic resistance is considered to be a major threat in the treatment of DFIs (Lipsky, 2007). Carriage of MDR microorganisms is mostly due to inappropriate antibiotic treatment, reduced antibiotic concentration in the tissue, chronic course of the wound, reduced effect of antibiotics in the wound environment and frequent hospital admission of DFU patients, where they are likely to be exposed to MDR organisms (Hartemann-Heurtier et al., 2004; Ozer et al., 2010). Available reports suggest that inadequate selection and abuse of antibiotics may lead to the development of resistance in various other bacteria and may make the treatment of bacterial infections more difficult (Kolár et al., 2001; Sáenz et al., 2004; Lipsky, 2007; Qing et al., 2012; Falcone et al., 2015). The exchange of resistance genes between bacterial chromosome and the plasmid (s) and their integration into particular genetic elements, called integrons, play a major role in acquisition and dissemination of resistance genes (Stokes and Hall, 1989). Additionally, natural transformation has the potential to promote exchange of DNA among taxonomically diverse bacteria (Ochman et al., 2000). Integron is found either as part of transposons or independently on several groups of broad-host-range plasmids. Different classes of integrons are distinguished by their highly conserved class-specific integrase genes and their overall genetic structure (Hall et al., 1999; Mazel, 2006). Till date, five classes of integrons (classes 1, 2, 3, 4, 5) with different *Int* genes have been reported (Mazel, 2006). Gene cassettes integrated into class 1 integrons in clinically important bacteria most commonly carry antibiotic resistance genes, and correlate with host bacterial resistance (El-Sokkary and Abdelmegeed, 2015). The high prevalence of class 1 integrons is known in clinically significant bacterial isolates viz. *Escherichia* (Sáenz et al., 2004), *Klebsiella* (Girlich et al., 2000), *Salmonella* (Gebreyes and Altier, 2002), and *Serratia* species (Centrón and Roy, 2002). PCR amplification of highly conserved 5′ and 3′ sequences of integrons has proved very useful target in epidemiological surveys of bacterial antibiotic resistance (White et al., 2001; Chang et al., 2009).

β-lactamase-producing bacteria (including those producing extended-spectrum β-lactamases), vancomycin-resistant enterococci (VRE), and methicillin-resistant *S. aureus* (MRSA) are important nosocomial pathogens in India and other parts of the world (Mehrotra et al., 2000; Jia et al., 2014; Mobarak-Qamsari et al., 2014). As such the presence of β-lactamase, ESBL-producing, VRE, and MRSA in DFUs seems crucial (Bradford, 2001; Sáenz et al., 2004). Several researchers have reported antibiotics resistant bacteria from DFUs but there is a paucity of ESBL, VRE, and MRSA data particularly in India (Shankar et al., 2005; Gadepalli et al., 2006; Shakil and Khan, 2010). β-lactamases are remarkably diversified due to their continuous mutation (Dallenne et al., 2010). Different types of β-lactamases have been described during the 1990s, TEM- and SHV-type ESBLs were dominant (Bradford, 2001). During the past decade, rapid and massive spread of CTX-M-type of ESBLs have been reported (Sana et al., 2011). These enzymes are now the most prevalent ESBLs in Enterobacteriaceae and also in *Pseudomonas* spp. and *Acinetobacter baumannii* (Bradford, 2001; Paterson and Bonomo, 2005) in Europe and other parts of the world (Coque et al., 2008).

Incidence of antibiotics resistance is becoming a serious problem in India, especially with the DFUs patients where no systematic study has been made to unravel the occurrence of MDR bacteria and/or the genetic basis of resistance in these bacteria. Infection in DFUs with MDR bacteria is known to increase the duration of hospital stay, cost of management as well as morbidity and mortality. Henceforth, early diagnosis of microbial infections and appropriate antibiotic therapy are needed to avoid further complications. In the present study, an attempt was made to determine the bacterial prevalence and reveal the genetic basis of MDR in bacteria isolated from DFUs.

## Materials and methods

### Patients and sample collection

This study was conducted in the School of Biotechnology in collaboration with the Departments of Endocrinology and Metabolism, and General Surgery, Sir Sunderlal Hospital, Institute of Medical Sciences, Banaras Hindu University, Varanasi. In total, 116 DFUs patients visiting Sir Sunderlal Hospital between January 2010 and October 2011 were examined and 42 patients suffering from severe DFUs (grades III to V) were included in the study. The grading of foot ulcers was done according to Wagner (1981) (grade 0- hyperkeratosis; grade I -superficial ulcers; grade- II deep ulcers; grade- III tendonitis, osteomyelitis, cellulites, or abscess; grade- IV gangrene of a toe or forefoot; and grade -V massive gangrene of the whole foot). The study was approved by the Institutional Ethics Committee (Ref. No. Dean/2009-10/555 dated July 11, 2009).

For sample collection, each DFU was cleaned with sterile saline and thereafter superficial swab sample was collected from the center of ulcer by applying a sterile cotton-tipped applicator. Deep tissue samples were obtained from the ulcer using a sterilized punch biopsy needle (6 mm) under local anesthesia after washing the wound with sterile physiological saline. Of two swab and tissue samples collected from DFU of each patient, one was used for detecting aerobic and anaerobic bacteria through *in vitro* culture, the second tissue samples were used for detecting anaerobes (*Bacteroides* spp., *Peptostreptococcus productus* and *Clostridium* spp.) by PCR in a culture-independent approach. Briefly, total genomic DNA of tissue samples was extracted employing a Fast Tissue PCR Kit following the instructions of the manufacturer (MBI Fermentas, USA). Genus specific primers of *16S rRNA* gene (Table 1) corresponding to *Bacteroides, P. productus*, and *Clostridium* were used for the amplification of desired amplicon in PCR assay (Rekha et al., 2006). For *in vitro* culture all the specimens were transported by sterile containers. Written consent of all the patients was taken before the collection of swab/tissue.

**Table 1:** **Primers used for amplification of different genes**.

**Genes**	**Primer sequence (5′ → 3′)**	**Amplicon size (bp)**	**References**
Class 1 integron	F CS: GGCATCCAAGCAGCAAG R CS: AAGCAGACTTGACCTGA	Variable	Lévesque et al., 1995
*aad*A1	F: GCACGACGACATCATTCCG R: ACCAAATGCGGGACAACG	300	Drum, 2003
*aad*B	F: ACGCAGGTCACATTGATAC R: CGGCATAGTAAGAGTAATCC	400	
*dhfr*V	F: CGATGTTTGATGTTATGG R: TGTTTCTCTGTAAATCTCCC	400	
*dhfr*XII	F: CGAACCGTCACACATTGG R: GCATAAACGGAGTGGGTG	300	
*bla*_TEM_	F: CATTTCCGTGTCGCCCTTATTC R: CGTTCATCCATAGTTGCCTGAC	800	Dallenne et al., 2010
*bla*_SHV_	F: AGCCGCTTGAGCAAATTAAAC R: ATCCCGCAGATAAATCACCAC	713	
*bla*_OXA_	F: GGCACCAGATTCAACTTTCAAG R: GACCCCAAGTTTCCTGTAAGTG	564	
*bla*_CTX−M−gp1_	F: TTAGGAARTGTGCCGCTGYA R: CGATATCGTTGGTGGTRCCAT	688	
*bla*_CTX−M−gp2_	F: CGTTAACGGCACGATGAC R: CGATATCGTTGGTGGTRCCAT	404	
*bla*_CTX−M−gp9_	F: TCAAGCCTGCCGATCTGGT R: TGATTCTCGCCGCTGAAG	561	
*vanA*	F: GGGAAAACGACAATTGC R: GTACAATGCGGCCGTTA	732	Jia et al., 2014
*vanB*	F: CATCGCCGTCCCCGAATTTCAAA R: GATGCGGAAGATACCGTGGCT	297	
*mecA*	F: CCAACTGTCGTAGTCGAAACC R: CTAAGGCACGTCAAAAATGGT	145	Mehrotra et al., 2000
*femA*	F: AAAAAAGCACATAACAAGCG R: GATAAAGAAGAAACCAGCAG	173	
*16S rRNA* (*Bacteroides* spp.)	F: GGG GTT CTG AGA GGA AG R: ACCCCCCATTGTACCAC	950	Rekha et al., 2006
*16S rRNA* (*Peptostreptococcus productus*)	F: GGTGCCGCAGTAAACACAATAGT R: AAGGCCCGGGAACGTATTCA	539	
*16S rRNA* (*Clostridium* spp.)	F: CTCAACTTGGGTGCTGCATTT R: ATTGTAGTACGTGTGTAGCCC	619	
*16S rRNA*	F: AGAGTT TGA TYM TGG CTC AG R: CTACGGCTACCTTGTTACGA	1500	Singh et al., 2009

*F, Forward; R, Reverse*.

### Isolation and identification of aerobic/anaerobic bacteria

Initially, samples were gently macerated and fixed for Gram staining. Samples were quickly examined for the types of bacteria (Gram positive/Gram negative) for empirical therapy. For isolation, a direct smear was made from each sample (swab and biopsy) and plated directly onto appropriate aerobic and anaerobic planting media such as blood agar, MacConkey agar, Chocolate agar, and Columbia blood agar. The plates were immediately transferred to an aerobic or anaerobic jar and incubated at 35°C for 24 and 48 h, respectively. The plates were examined after 24–48 h of incubation and distinct colonies appearing on each plate were picked up and restreaked on respective media. Isolates with distinct morphotypes from each plate were selected for further characterization. Tentative identification of different aerobic isolates was made on the basis of Gram's staining and morphological characters as well as biochemical tests namely, catalase, urease, Simmons citrate utilization and methyl red as per the standard method (Kimberley and Elsa, 2003). For the detection of anaerobic bacteria culture-independent approach was employed as described above.

### Antibiotic susceptibility testing

Antibiotic susceptibility test of different strains was done by the disc diffusion using the Kirby-Bauer method (Bauer et al., 1966). Eighteen antibiotics belonging to nine different classes i.e., (a) cephalosporins: cefazolin 30 μg (1st generation), cefoxitin 30 μg (2nd generation), cefoperazone 75 μg (3rd generation) and cefepime 30 μg (4th generation), (b) aminoglycosides: gentamicin 10 μg, amikacin 30 μg, kanamycin 30 μg, streptomycin 20 μg, and spectinomycin 100 μg, (c) penicillins: methicillin 30 μg, piperacillin/tazobactam 100/10 μg, and ampicillin 10 μg, (d) lincosamide: clindamycin 2 μg, (e) tetracycline: tetracycline 30 μg, (f) carbapenem: meropenem 10 μg, (g) dihydrofolate reductase inhibitor: trimethoprim 20 μg, and (h) folate pathway inhibitor: co-trimoxazole 25 μg, (i) glycopeptide: vancomycin 30 μg were selected according to published recommendations and their widespread use in the treatment of various diseases (Gadepalli et al., 2006). The antibiotic disc was purchased from HiMedia Laboratories (Mumbai, India) and susceptibility profile (intermediate or susceptible) against antibiotics was deduced from the manual supplied by the manufacturer. Unless otherwise stated intermediate susceptible isolates were counted as resistant to all the agents tested. Interpretation of result is based according to the Clinical and Laboratory Standards Institute (CLSI)^1^ guidelines 2010. Herein, MDR was defined for an isolate showing resistance to antibiotics belonging to three or more classes.

### Amplification and sequencing of 16S rDNA

Genomic DNA was extracted employing DNEasy extraction kit (Qiagen, Germany) according to the instructions of manufacturer. 16S rDNA (1.5 kb) was amplified using universal primers (Table 1). The PCR reaction mix (50 μl) included; 1.5 U of *Taq* DNA polymerase (Bangalore Genei, India), 1 X PCR assay buffer with 1.5 mM MgCl_2_, 50 pmol of each forward and reverse primers (Integrated DNA Technologies, USA), 125 μM of each dNTPs (Bangalore Genei, India) and 50 ng template DNA. Thermal cycle was set as described earlier (Singh et al., 2009). Sequencing of amplified product was done on commercial basis from Chromous Biotech Pvt. Ltd. (Bangalore, India). All the sequences were matched against nucleotide sequences present in GenBank using the BLASTn program to identify the most similar 16S rDNA (Altschul et al., 1997; www.ncbi.nlm.nih.gov/blast). The 16S rDNA sequences of 38 bacterial strains have been deposited in the GenBank data base and accession numbers have been obtained.

### Phylogenetic analysis

16S rDNA sequences of 37 isolates of this study and 109 sequences of strains reported from different parts of the world were used to construct phylogenetic trees. Multiple alignments of sequenced nucleotides were carried out using ClustalW2 (version 2.0.10). Neighbor joining tree was constructed in MEGA 5.0 (Tamura et al., 2011) using bootstrapping at 1000 bootstrap trials with the two-parameter model of Kimura.

### Amplification of class 1 integron and associated gene cassettes

The variable region of class 1 integron was amplified using forward primer 5′-GGC ATC CAA GCA GCA AG-3,′ and the reverse primer 5′-AAG CAG ACT TGA CCT GA-3′ as described earlier (Lévesque et al., 1995). Purified genomic DNA of the bacteria was used as template. Five microliters of the amplified PCR product was electrophoresed on a 1% agarose gel in TAE buffer (40 mM Tris/acetate (pH 8.0), 1 mM EDTA) containing ethidium bromide (0.5 μg ml^−1^). Similarly, other genes namely *aad*A1, *aad*B, *dhfr*V, and *dhfr*XII within class 1 integron were amplified using specific primers (Table 1).

### Restriction digestion of amplified class 1 integron

Amplified product of variable region of class 1 integron (1.6 kb) from *Pseudomonas* spp. DF5TC, *Cronobacter* spp. DF52TC, *Alcaligenes* spp. DF19TB, *Stenotrophomonas* spp. DF3SA, *K. pneumoniae* DF12SA, *E. coli* DF30TA, *Providencia* spp. DF1SB, *Alcaligenes* spp. DF43SB, *S. flexneri* DF1TA, *S. aureus* DF8TA, *Enterococcus* spp. DF5SB, and *Enterococcus* spp. DF16SA was purified and digested with *Alu*I and *Rsa*I following the instructions of the manufacturer (New England BioLabs). Restriction digestion was done in a final volume of 25 μl containing 1 x restriction enzyme buffer, 0.30 μl (3.0 U) restriction enzyme and 15 μl PCR product. After mixing, samples were incubated for 6 h in a water bath preset at 37°C. Reaction was terminated by heat inactivation of restriction enzymes at 70°C for 20 min. The digested products were run in a 3% agarose gel at 100 V in TAE buffer for 4–5 h. Cluster analysis of restriction fragment length polymorphism (RFLP) types was performed by the unweighted pair-group method with arithmetic means using Quantity One 1-D Analysis Software, version 4.4 (BioRad).

### Sequencing of class 1 integron

Variable region of class 1 integron (1.6 kb) amplified from *K. pneumoniae* DF12SA was purified by Invitrogen kit (Invitrogen Corpn, USA) following the instructions of the manufacturer. Purified products were sequenced on commercial basis from Chromous Biotech Pvt. Ltd. (Bangalore, India). The sequence was matched against nucleotide sequences present in GenBank using the BLASTn program (Altschul et al., 1997) at website www.ncbi.nlm.nih.gov/blast. Variable region of class 1 integron sequence of *K. pneumoniae* DF12SA was submitted to NCBI and accession number was obtained (HQ114261).

### Multiplex PCR

Multiplex PCR for *bla*_TEM_, *bla*_SHV_*, bla*_OXA_, and *bla*_CTX−M−gp1_, *bla*_CTX−M−gp2_, and *bla*_CTX−M−gp9_ genes was performed by multiplexing of primers (Table 1) of respective genes. Thermal cycles for the amplification were set as: initial denaturation for 10 min at 94°C, 30 cycles of 40 s at 94°C, 40 s at 60°C, and 1 min at 72°C and final extension for 7 min at 72°C (Dallenne et al., 2010). *E. coli* ATCC strain 25922 and *K. pneumoniae* ATCC strain 700603 (HiMedia, Mumbai, India) were used as negative and positive controls respectively for the amplification of ESBL genes.

*S. aureus, S. haemolyticus*, and *Enterococcus* strains were screened for the presence of *mecA* (oxacillin resistance), *femA* (methicillin resistance), *vanA*, and *vanB* (vancomycin resistance) genes using respective primers (Table 1) as described in previous studies (Arakere et al., 2005; Jia et al., 2014).

## Results

### Isolation and initial characterization of bacteria

Altogether 142 aerobic bacteria (69 from swabs and 73 from tissues) belonging to 17 genera were successfully isolated from swab and deep tissue of DFUs (Wagner's grade III to V) of 42 diabetic patients. Tentative identification of all the 142 aerobic isolates was made on the basis of growth features on specific media, morphological characters, Gram's staining and biochemical tests. Of these, 53 (37.32%) belonged to Gram-positive, and 89 (62.67%) to Gram-negative group. It was noted that bacteria such as *Enterococcus* spp., *E. coli, Staphylococcus* spp., *Alcaligenes* spp., *Pseudomonas* spp. and *Stenotrophomonas* spp. were the most common in DFUs, the percentage distribution being 47.61, 35.71, 33.33, 30.95, 30.95, and 30.95, respectively. However, bacteria such as *Cronobacter* spp. and *Achromobacter xylosoxidans* were present in 2.38% of DFUs (Table 2).

**Table 2:** **Tentative identification of aerobic bacteria isolated from swab and tissue samples of DFUs**.

**Bacteria**	**Swab**	**Tissue**	**No patients (%)**
**GRAM-POSITIVE AEROBES**			
*Enterococcus* spp.	10	7	20^*^ (47.61)
*Enterococcus faecalis*	1	3	
*Enterococcus raffinosus*	2	2	
*Enterococcus gilvus*	1	0	
*Staphylococcus aureus*	7	4	14^*^ (33.33)
*Staphylococcus haemolyticus*	2	4	
*Corynebacterium* spp.	3	1	4 (9.52)
*Paenibacillus* spp.	1	2	3 (7.14)
*Exiguobacterium mexicanum*	0	3	3 (7.14)
**GRAM-NEGATIVE AEROBES**			
*Escherichia coli*	7	9	15^*^ (35.71)
*Alcaligenes* spp.	8	6	13^*^ (30.95)
*Alcaligenes faecalis*	0	2	
*Stenotrophomonas* spp.	7	6	13 (30.95)
*Pseudomonas* spp.	5	2	13^*^(30.95)
*Pseudomonas fluorescens*	1	5	
*Pseudomonas stutzeri*	1	1	
*Klebsiella pneumonia*	3	5	8 (19.04)
*Providencia* spp.	3	5	7^*^ (16.66)
*Shigella flexneri*	4	2	6 (14.28)
*Serratia* spp.	1	2	3 (7.14)
*Psychrobacter faecalis*	1	1	2 (4.76)
*Cronobacter* spp.	0	1	1 (2.38)
*Achromobacter xylosoxidans*	1	0	1 (2.38)
	69	73	

* *Certain bacteria present both in swab and tissue. Value in parenthesis represents percentage*.

**Figure 1:**
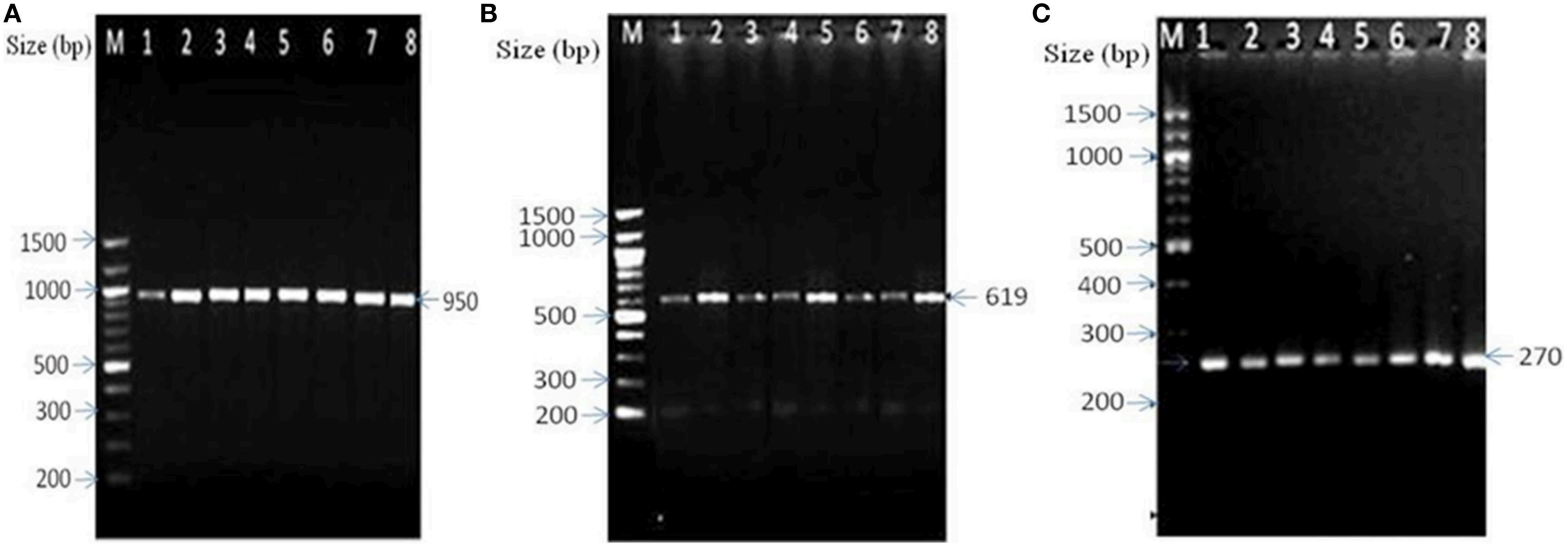
**Amplification of genus specific amplicon of anaerobic bacteria by PCR**. **(A)** Amplified amplicon of *Bacteroides* sp., lanes 1–8: 1-DF4, 2-DF5, 3-DF8, 4-DF13, 5-DF18, 6-DF20, 7-DF22, and 8-DF24. **(B)** *Clostridium* sp., lanes 1–8: 1-DF1, 2-DF3, 3-DF4, 4-DF7, 5-DF9, 6-DF12, 7-DF14, and 8-DF19, and **(C)** *Peptostreptococcus productus*, Lanes1–8:1-DF1, 2-DF4, 3-DF5, 4-DF6, 5-DF11, 6-DF13, 7-DF15, and 8-DF24. M- 100 bp ladder (New England Biolabs, USA).

Cultivation-independent approach mainly based on the amplification of genus-specific *16S rRNA* gene showed amplification of the 16S rDNA fragment specific to *Bacteriodes* spp., *P. productus*, and *Clostridium* spp. in DFUs of 29 (69.04%) patients (Table 3, Figures 1A–C). Of the 29 DFUs, 18 had only one of the above three isolates but 11 contained two or three isolates (Table 3). On the other hand, routine culture method showed the presence of anaerobes in DFUs of 12 (28.57%) patients (Table 3). Furthermore, majority of DFUs showed the presence of only one type of anaerobe contrary to culture-independent method where three genera namely *Clostridium* spp. *P. productus*, and *Bacteriodes* spp. were detected in DFU of four patients (Table 3). Among all the anaerobes detected by PCR, the prevalence of *Clostridium* was maximum, being present in DFUs of 19 subjects (45.23%) followed by *P. productus* in 13 (30.95%) and *Bacteroides* in 12 (28.57%). Further analysis of bacterial prevalence showed as high as eight species of bacteria (6 aerobic and 2 anaerobic) in DFU of one patient (DF43). Average 3.38 bacteria were detected from the DFU of each patient.

**Table 3:** **Anaerobic bacteria detected by culture-dependent/independent approach from deep tissue of DFU patients**.

**Anaerobic bacteria**	**Detection of anaerobes by culture- dependent approach**	**No of patients**	**Detection of anaerobes using culture- independent approach**	**No of patients (%)**
**GRAM-NEGATIVE ANAEROBES**				
*Bacteroides* spp.	DF20, DF24, DF43	3 (7.14)^*^	DF8, DF18, DF38	3 (7.14)
**GRAM-POSITIVE ANAEROBES**				
*Peptostreptococcus productus*	DF1, DF13, DF36	3 (7.14)	DF6, DF11, DF15	3 (7.14)
*Clostridium* spp.	DF4, DF14, DF29, DF32, DF40	5 (11.90)	DF3, DF7, DF9, DF12, DF14, DF19, DF23, DF29, DF32, DF34, DF39, DF44	12 (28.57)
**ANAEROBES COMBINATION IN DFUs TISSUE**				
*Clostridium* spp.	0	0	DF20	1 (2.38)
*Bacteroides* spp.				
*Clostridium* spp. *Peptostreptococcus productus*	DF27	1 (2.38)	DF1, DF36,	2 (4.76)
*Bacteroides* spp. *Peptostreptococcus productus*	0	0	DF5, DF13, DF22, DF43	4 (9.52)
All above three isolates	0	0	DF4, DF24, DF27, DF40	4 (9.52)
		12		29

* *Value in parenthesis represents percentage*.

*DF, diabetic foot. Number represents serial number of patients*.

### Antibiotic susceptibility test, identification of MDR isolates using *16S rRNA* gene sequencing and phylogenetic analysis

Antibiotic susceptibility test of 142 aerobic bacteria revealed that 38 (26.76%) were resistant to antibiotics belonging to three or more classes. Henceforth, these 38 isolates were designated as MDR bacteria. Prevalence of resistance to different antibiotics among the isolates was; cefazolin (65.78%), cefoxitin (73.68%), cefoperazone (34.21%), cefepime (68.42%), gentamycin (94.73%), amikacin (50%), kanamycin (92.10%), streptomycin (65.78%), spectinomycin (73.52%), piperacillin/tazobactam (26.31%), ampicillin (89%), clindamycin (34.21%), tetracycline (60.52%), meropenem (76.31%), trimethoprime (63.15%), and co-trimoxazole (76.31%). 71.4% of *Enterococcus* spp. were resistant to vancomycin whereas all the *Staphylococcus* spp. were resistant to methicillin. Among all the MDR isolates, *K. pneumoniae* strain DF12SA showed resistance to as many as 15 antibiotics and *E. coli* strain DF39TA and *E. faecalis* DF43SA to 14 antibiotics.

As 38 isolates exhibited MDR property, their identification was confirmed by 16S rDNA sequencing. These isolates belonged to different species/strains of *Enterococcus, Staphylococcus, Corynebacterium, Paenibacillus, Exiguobacterium, Escherichia, Alcaligenes, Stenotrophomonas, Pseudomonas, Klebsiella, Providencia, Shigella, Serratia, Psychrobacter, Cronobacter*, and *Achromobacter* (Table 4). The nucleotide sequences of the 16S rDNA of all the isolates were submitted to NCBI and accession numbers were obtained (Table 4).

**Table 4:** **Occurrence of class-1 integron and β- lactamase genes in MDR bacteria**.

**Bacteria**	Accession No^*^	**Resistance phenotype**	**Class 1 integron size (kb)**	**Gene cassette (s)**	**β- lactamase genes**
*Enterococcus* sp. DF5SB	HQ163798	GEN, KAN, STR, SPX, AMP, TET, TMP, CTZ, VAN	1.6	*dhfrAXVII*, a*adAV*	*bla*_TEM_, *vanA, vanB*
*Enterococcus* sp. DF19SA	HQ163796	CFP, FEP, GEN, AMK, KAN, AMP, TET, CTZ, VAN	NA	NA	*bla*_TEM_, *vanA, vanB*
*Enterococcus* sp. DF16SA	HQ163795	CXT, GEN, AMK, KAN, STR, SPX, AMP, MEM, TMP, CTZ, VAN	1.6	*dhfrAXVII, aadAV*	*bla*_TEM,_*vanA, vanB*
*Enterococcus faecalis* DF30TB	JN642255	CXT, FEP, GEN, KAN, AMP, TET, MEM, CTZ, VAN	NA	NA	*bla*_TEM_, *vanB*
*Enterococcus faecalis* DF43SA	JN642254	CEZ, FEP, GEN, AMK, KAN, STR, SPX, PTZ, AMP, TET, MEM, TMP, CTZ, VAN	4.0 and 2.5	*aadB, dhfrV*	*bla*_TEM_*, vanB*
*Enterococcus raffinosus* DF11TA	JN642264	CEZ, CXT, GEN, KAN, CLD, TET, MEM, CTZ	NA	NA	NA
*Enterococcus gilvus* DF11SD	JN642251	CXT, FEP, GEN, KAN, STR, SPX, AMP, CLD, MEM	0.5	*aadB*	*bla*_TEM,_
*Staphylococcus aureus* DF8TA	JN642261	CXT, GEN, AMK, KAN, STR, SPX, PTZ, AMP, TET, TMP, CTZ, MTC	1.6	*dhfrAXVII, aadAV*	*bla*_TEM,_*mecA, femA*
*Staphylococcus haemolyticus* DF5TA	HQ163797	CEZ, CXT, FEP, AMP, TET, MEM, TMP, CTZ, MTC	0.3	*dfhrXII*	*bla*_TEM_,_,_*mecA, femA*
*Corynebacterium* sp. DF26SB	JN642257	CXT, FEP, GEN, AMK, KAN, AMP, CLD, TET, MEM	NA	NA	*bla*_TEM_, *bla*_OXA_
*Paenibacillus* sp. DF9SA	JN642249	CXT, GEN, KAN, STR, SPX, PTZ, AMP, CLD, TET	0.9	*aadB*	*bla*_TEM_
*Exiguobacterium mexicanum* DF43TB	JN642268	CEZ, FEP, GEN, AMK, KAN, STR, SPX, AMP, TET, MEM, TMP, CTZ	2.5	*dfhrXII, aadB, dhfrV*	*bla*_TEM_, *bla*_OXA_
*E. coli* DF30TA	JN642269	CEZ, CXT, GEN, FEP, AMK, KAN, STR, SPX, AMP, MEM, TMP, CTZ	1.6	*dhfrAXVII, aadAV*	*bla*_TEM_, *bla*_SHV,_*bla*_CTX−M−gp1_
*E. coli* DF30TD	JN642266	CEZ, CXT, CFP, FEP, GEN, AMK, AMP, PTZ, CLD	NA	NA	*bla*_TEM_, *bla*_SHV_, *bla*_CTX−M−gp1_
*E. coli* DF39TA	HQ163793	CEZ, CXT, CFP, FEP, GEN, AMK, KAN, STR, SPX, AMP, TET, MEM, TMP, CTZ	2.4	*aadB, dhfrV, aadA1*	*bla*_TEM_, *bla*_SHV_, *bla*_OXA_, *bla*_CTX−Mgp1_
*Alcaligenes* sp. DF3TA	HQ163791	CXT, CFP, KAN, AMP, CLD, TET, PTZ, MEM	NA	NA	*bla*_TEM_
*Alcaligenes* sp. DF18SC	HQ163792	CFP, CEZ, CLD, GEN, KAN, STR, SPX, MEM, TMP, CTZ	1.2	*aadA1*	NA
*Alcaligenes* sp. DF19TB	JN642262	CEZ, CXT, FEP, GEN, KAN, STR, SPX, AMP, TMP, CTZ	1.6 and 0.5	*dhfrAXVII, aadAV, aadB*	*bla*_TEM_, *bla*_OXA_
*Alcaligenes* sp. DF29SB	JN642259	CEZ, CXT, FEP, GEN, KAN, STR, SPX, AMP, PTZ, CLD, MEM, TMP, CTZ	1.7 and 0.7	*aadB, dhfrV*	*bla*_TEM_, *bla*_SHV_*, bla*_OXA_
*Alcaligenes* sp. DF34SB	JX869134	CEZ, CXT, GEN, AMK, KAN, AMP, TET, MEM	NA	NA	*bla*_TEM_
*Alcaligenes* sp. DF36TC	HQ163794	FEP, GEN, KAN, STR, SPX, MEM, TMP, CTZ	0.85	*aadB*	*bla*_TEM_, *bla*_OXA_
*Alcaligenes* sp. DF43SB	JN642256	CEZ, CXT, GEN, KAN, STR, SPX, AMP, CLD, TET, TMP, CTZ	1.6	*dhfrAXVII, aadAV*	*bla*_TEM_, *bla*_OXA_, *bla*_CTX−M−gp1_
*Alcaligenes faecalis* DF45TB	JX869135	CEZ, CXT, FEP, GEN, AMK, KAN, CLD, AMP, MEM	NA	NA	*bla*_OXA_, *bla*_CTX−M−gp1_
*Stenotrophomonas* sp. DF3SA	JN642253	CEZ, CXT, FEP, GEN, AMK, KAN, STR, SPX, AMP, PTZ, TET, TMP, CTZ	1.6	*dhfrAXVII, aadAV*	*bla*_TEM_, *bla*_OXA_, *bla*_CTX−M−gp1_
*Stenotrophomonas* sp. DF9SD	JX869133	CEZ, CFP, FEP, GEN, KAN, AMP, TET, MEM, TMP, CTZ	0.3	*dhfrXII*	*bla*_TEM_, *bla*_CTX−M−gp2_
*Stenotrophomonas* sp. DF17TA	JN642265	CEZ, CXT, FEP, GEN, AMP, CLD, TET, MER	NA	NA	*bla*_TEM,_ *bla*_CTX−M−gp2_
*Pseudomonas* sp. DF5TC	HQ163790	CEZ, CXT, FEP, GEN, KAN, STR, SPX, CLD, MEM, TMP, CTZ	1.6	*dhfrAXVII, aadAV*	*bla*_TEM_, *bla*_OXA_, *bla*_CTX−M−gp1_, *bla*_CTX−M−gp9_
*Pseudomonas fluorescens* DF7SB	JN642246	CXT, FEP, GEN, AMK, KAN, STR, SPX, AMP, TET, MEM, TMP, CTZ	2.4	*aadB, dhfrV, aadA1*	*bla*_TEM_, *bla*_SHV_, *bla*_OXA_
*Pseudomonas fluorescens* DF41TB	JN642252	CFP, FEP, GEN, KAN, AMP, PTZ, MER, TET, CTZ	NA	NA	*bla*_TEM_, *bla*_OXA_, *bla*_CTX−M−gp1_
*Pseudomonas stutzeri* DF1SA	JN642263	CEZ, CXT, GEN, AMK, KAN, STR, SPX, AMP, MEM	1.2	*aadA1*	*bla*_TEM_
*Klebsiella pneumoniae* DF12SA	HQ114261	CEZ, CXT, CFP, FEP, GEN, AMK, KAN, STR, SPX, PTZ, AMP, TET, MEM, TMP, CTZ	1.6	*dhfrAXVII, aadAV*	*bla*_TEM_, *bla*_CTX−M−gp9_
*Providencia* sp. DF1SB	HQ163789	CEZ, CXT, CFP, FEP, GEN, KAN, STR, SPX, AMP, MEM, TMP, CTZ	1.6	*dhfrAXVII, aadAV*	*bla*_TEM_, *bla*_OXA_, *bla*_CTX−M−gp1_
*Providencia* sp. DF14SA	JN642260	CEZ, CXT, FEP, GEN, KAN, STR, SPX, AMP, CLD, TMP, CTZ	2	*dfhrXII, aadB, dhfrV*	*bla*_TEM_, *bla*_OXA_
*Shigella flexneri* DF1TA	JN642248	CEZ, CXT, FEP, GEN, KAN, STR, SPX, AMP, TET, MEM, TMP, CTZ	1.6	*dhfrAXVII, aadAV*	*bla*_TEM_ *bla*_OXA_, *bla*_CTX−M−gp1_
*Serratia* sp. DF15SB	JN642247	CEZ, CXT, CFP, FEP, GEN, AMK, KAN, STR, SPX, AMP, MEM,TMP, CTZ	2.4 and 1.4	*aadB, dhfrV, aadA1*	*bla*_TEM_, *bla*_CTX−M−gp1_, *bla*_CTX−M−gp2_
*Psychrobacter faecalis* DF30TC	JN642258	CEZ, FEP, GEN, AMK, KAN, AMP, TET, MER	NA	NA	*bla*_TEM_, *bla*_OXA_
*Cronobacter* sp. DF52TC	JN642250	CXT, CFP, GEN, AMK, KAN, STR, SPX, AMP, TET, MEM, TMP, CTZ	1.6	*dhfrAXVII, aadAV*	*bla*_TEM_
*Achromobacter xylosoxidans* DF33SA	JN642267	CXT, CFP, FEP, GEN, AMK, KAN, PTZ, AMP, MEM	NA	NA	*bla*_TEM_, *bla*_SHV_, *bla*_OXA_

* *Accession no. based on 16S r RNA sequence*.

*AMK, Amikacin; AMP, Ampicillin; CEZ, cefazolin; CFP, Cefoperazone; FEP, Cefepime; CLD, Clindamycin; CTZ, Co-trimoxazole; CXT, cefoxitin; GEN, Gentamycin; KAN, Kanamycin; MEM, Meropenem; MTC, methicillin; PTZ, Piperacillin/tazobactam; SPX, Spectinomycin; STR, Streptomycin; TET, Tetracycline; TMP, Trimethoprim; VAN, Vancomycin; R, resistant; S, susceptible; NA, no amplification of any gene*.

**Figure 2:**
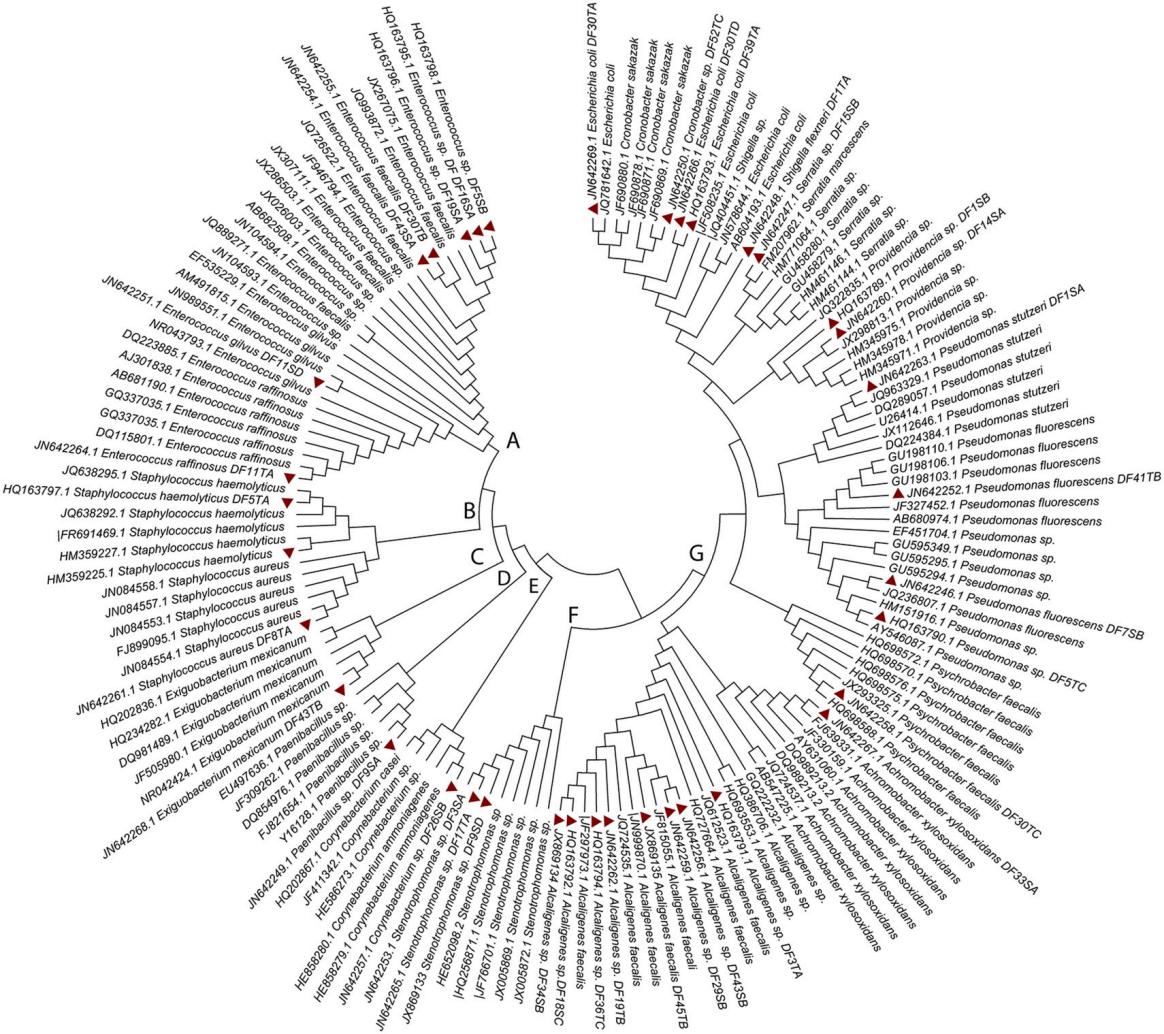
**Phylogenetic analysis based on the sequences of 16S ***rRNA*** gene sequence of 37 DFUs isolates and 110 sequences retrieved from NCBI**. The evolutionary history was inferred using the Neighbor-joining method. The bootstrap consensus tree inferred from 1000 replicates is taken to represent the evolutionary history of the taxa analyzed. Branches corresponding to partitions reproduced in less than 50% bootstrap replicates are collapsed. The tree is drawn to scale, with branch lengths in the same units as those of the evolutionary distances used to infer the phylogenetic tree. The evolutionary distances were computed using the Kimura 2-parameter method and are in the units of the number of base substitutions per site. The accession number of each strain is mentioned in tree. Thirty eight isolates identified by *16S rDNA* sequencing are highlighted in red.

Phylogenetic analysis based on 16S rDNA sequences of all the identified isolates of this study and 110 sequences from the isolates of different parts of the world showed separate clustering of our isolates. Additionally, significant diversity was evident among these isolates. It is evident from the tree (Figure 2) that all the isolates could be grouped in seven clusters. Clusters A, B, C, D, E, F, and G included 28, 12, 6, 6, 6, 8, and 81 isolates, respectively. Furthermore, phylogenetic analysis suggested a high degree of relatedness between certain isolates of this study with strains of bacteria reported from different parts of the world.

### Occurrence of class 1 integrons

PCR amplification of variable regions of class 1 integrons repeatedly showed variable sizes (0.3–4.0 kb) in different isolates. Notably, the size of amplicon showed significant variations among different species/strains. Among 26 integron positive isolates, 13 types of gene cassette with size of 0.3, 0.5, 0.7, 0.85, 0.9, 1.2, 1.4, 1.6, 1.7, 2.0, 2.4, 2.5, and 4 kb were observed (Table 4). However, 12 isolates namely *Pseudomonas* spp. DF5TC, *Cronobacter* spp. DF52TC, *Alcaligenes* spp. DF19TB, *Stenotrophomonas* spp. DF3SA, *K. pneumoniae* DF12SA, *E. coli* DF30TA, *Providencia* spp. DF1SB, *Alcaligenes* spp. DF43SB, *S. flexneri* DF1TA, *S. aureus* DF8TA, *Enterococcus* spp. DF5SB, and *Enterococcus* spp. DF16SA showed the presence of only one amplicon of 1.6 kb size. Sequencing of amplified variable region of class 1 integron (1.6 kb) of *K. pneumoniae* DF12SA was done and its analysis showed 99% homology with class 1 integron sequence of *K. pneumoniae* (FJ876827), *E. coli* (HQ880272), *S. flexneri* (FJ895301), *Staphylococcus epidermidis* (AB291061), *P. aeruginosa* (DQ838665), and *Stenotrophomonas maltophilia* (GQ924479) available in NCBI GenBank. Additionally, varying region of class 1 integron of 1.6 kb size amplified from all the 12 isolates was digested with *Alu*I and *Rsa*I. Results of restriction analysis revealed identical banding pattern in all the 12 isolates.

**Figure 3:**
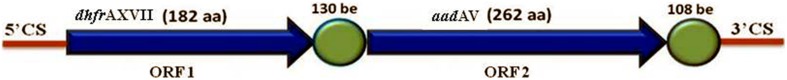
**Representation of class 1 integron (1.6 kb) organization in ***K. pneumoniae*** DF12SA**. Blue color indicates the gene, green color shows the base elements, and the red color represents 5'conserved segment (5′-CS) and 3′-CS of class 1 integron.

Sequencing of the 1.6 kb amplicon suggested presence of two ORFs (Figure 3). The first ORF (ORF1) was identified as *dhfr*AXVII which confers resistance to trimethoprim, and the second (ORF2) as *Aad*AV which confers resistance to streptomycin and spectinomycin. Gene cassettes *dhfr*XVII and *aadA*V were common in all the 12 isolates. In addition to *dhfr*XVII and *aadA*V, other gene cassettes namely, *aad*A1, *aad*B, *dhfr*V, and *dhfr*XII were detected in a number of isolates (Table 4). Altogether six genes encoding resistance to aminoclycoside (*aad*A1, *aad*B, and *aad*AV), and trimethoprim (*dhfr*V, *dhfr*XII, and *dhfr*XVII) were detected in different isolates. Interestingly, occurrence of class 1 integron in *E. mexicanum* DF43TB was also noted which appears to be a rare finding.

### Occurrence of *bla*_TEM_, *bla*_SHV_, *bla*_OXA_, *bla*_CTX-M_ (β-lactamase genes), and *mecA* (oxacillin), *femA* (methicillin) *vanA*, and *vanB* (vancomycin) resistance genes

All the 38 isolates showing MDR phenotype were also screened for the presence of cefotaxime hydrolyzing β-lactamase (CTX-M) by multiplex PCR. Fourteen isolates showed alleles encoding CTX-M enzymes belonging to the phylogenetic groups 1, 2, and 9 (CTX-M-gp1, CTXM-gp2, and CTX-M-gp9 related enzymes). Among these, nine isolates namely *E. coli* DF30TA, *E. coli* DF30TD, *E. coli* DF39TA, *Alcaligenes* spp. DF43SB, *A. faecalis* DF45TB, *Stenotrophomonas* spp. DF3SA, *P. fluorescens* DF41TB, *Providencia* spp. DF1SB, and *S. flexneri* DF1TA showed gene for CTX-M-gp1 enzyme only (Table 4). Two isolates namely *Stenotrophomonas* spp. DF9SD and *Stenotrophomonas* spp. DF17TA had the gene for CTX-M-gp2 type enzyme. Gene encoding CTX-M-gp9 enzyme was found in *K. pneumoniae* DF12SA. Both CTX-M-gp1 and CTX-M-gp2 were present in *Serratia* spp. DF15SB. Likewise, CTX-M-gp1 and CTX-M-gp9 were noted in *Pseudomonas* spp. DF5TC.

All the 38 MDR isolates were further screened for the presence of certain other β-lactamase genes viz. *bla*_TEM_, *bla*_SHV_, and *bla*_OXA_ by multiplex PCR. Among all the isolates amplification of *bla*_TEM_-like gene was attained in 34 isolates, *bla*_OXA_ in 18, and *bla*_SHV_ in 6 isolates (Table 4). Seventeen isolates were found positive for both *bla*_TEM_ and *bla*_OXA_ type β-lactamases (Table 4). *bla*_TEM_, *vanA*, and *vanB* were present in *Enterococcus* sp. DF5SB, *Enterococcus* spp. DF19SA, and *Enterococcus* sp. DF16SA whereas *bla*_TEM,_ *mecA*, and *femA* were present in *S. aureus* DF8TA and *S. haemolyticus* DF5TA. Distribution of various β-lactamases and methicillin, and vancomycin resistance genes in different isolates is presented in Table 4.

## Discussion

DFIs seem to be polymicrobial in nature as aerobic bacteria (*Enterococcus* spp., *E. coli, Staphylococcus* spp., *Alcaligenes* spp., *Pseudomonas* spp., and *Stenotrophomonas* spp.), and anaerobic bacteria (*Bacteroides* spp., *Clostridium* spp., and *Peptostreptococcus* spp.) were isolated from the severe DFUs (Wagner's grade, III-V) patients in this study. Our findings are similar to the study conducted by other groups wherein aerobic bacteria (*Staphylococcus* spp., *Streptococcus* spp., and Enterobacteriaceae), anaerobic bacteria (*Bacteroides* spp., *Clostridium* spp., and *Peptostreptococcus* spp.) and fungi (*Candida albicans*, and *Candida tropicalis*) were isolated from DFUs (Candel et al., 2003; Turhan et al., 2015). Enterococci are the most common cause of DFIs. Higher incidence of MDR enterococci in DFIs is expected in view of their well-documented role in several other infections. This is a tentative explanation, exact cause is not known to us. Though earlier studies showed Gram-positive aerobes as predominant in DFIs (Dang et al., 2003; Spichler et al., 2015), our findings revealed dominance of Gram-negative aerobic bacteria. The ratio of Gram-positive aerobes to Gram-negative aerobes was 1:1.67, which is contrary to earlier report (Tentolouris et al., 1999). The differences in the age, gender, ulcer grades, study setting, etc. in our study population as compared to other studies might be responsible for differences. However, our results are similar to the study conducted in other parts of India (Gadepalli et al., 2006) where Gram-negative bacteria were more common than Gram-positive bacteria in DFIs.

Conventional culture methods for anaerobes have been mostly used to identify bacteria in DFUs. Culture methods usually revealed a single organism (Shankar et al., 2005) and sometimes even failed to demonstrate organisms despite other clinical evidences of infection. However, in recent years PCR methods have made it possible to detect most species of pathogens in the wound in a matter of hours rather than days. We employed culture-independent method mainly based on PCR amplification of genus-specific amplicon (*16S rRNA*) and the results obtained were encouraging. Desired amplicons from a few anaerobic bacteria were successfully amplified using genus-specific primers. Herein, application of PCR assay using template DNA extracted from the tissue of DFUs allowed to gain a better insight of anaerobic bacteria as compared to culture-dependent method. This was evident from the fact that *Clostridium* was frequently identified in DFUs followed by *P. productus* and *Bacteroides* whereas certain other studies showed higher prevalence of *Peptostreptococcus* spp. (Colayco et al., 2002). It is concluded that PCR assay may be useful to unravel the complexity of bacterial occurrence especially anaerobes in DFUs of diabetic patients.

It was evident from the results that majority of the isolates were resistant to a number of antibiotics but certain isolates were sensitive to cefoperazone, piperacillin/tazobactam, and clindamycin. Altogether, our findings clearly suggest that the prevalence of MDR bacteria is fairly common in severe DFUs and support the findings of earlier studies (Hartemann-Heurtier et al., 2004; Gadepalli et al., 2006; Djahmi et al., 2013). It has been reported that about one-third of patients with a history of previous hospitalization for the same wound, and 25% of patients with osteomyelitis, had MDR bacteria in the specimens (Hartemann-Heurtier et al., 2004). Gadepalli et al. (2006) reported 44.7 and 56.0% β-lactamase-producing and methicillin resistant bacteria, respectively in DFUs from North India (New Delhi). Incidence of relatively higher frequency of antibiotic resistance in this study could be due to the fact that Sir Sunderlal Hospital of Banaras Hindu University, Varanasi, is a tertiary care hospital with widespread usage of broad spectrum antibiotics leading to selective survival advantage of bacteria. Additionally, increase in antibiotic resistance might be the result of irrational use of antibiotics, and horizontal transfer of antibiotic resistant genes among bacteria by mobile genetic elements including plasmids, transposons, and integrons (Domingues et al., 2012). Since a plasmid or transposon can carry several resistance determinants, simultaneous resistance to multiple antibiotics may be attained. It would have been worthwhile to isolate plasmid from different isolates as their presence cannot be ruled out.

Resistance to β-lactam antibiotics in Gram-negative bacteria is primarily mediated by β-lactamases, which hydrolyze the β-lactam ring and thus inactivate the antibiotic (Livermore and Woodford, 2006). Different β-lactamases have been described, but *bla*_TEM_, *bla*_SHV_, *bla*_OXA_, and *bla*_CTX−M_ like genes are the most predominant in Gram-negative bacteria (Livermore and Woodford, 2006; Ahmed et al., 2007; Shahi et al., 2013). Our findings are in agreement with above reports as β-lactamase genes conferring resistance to β-lactam antibiotics were noted in 35 out of 38 MDR isolates. Among the identified genes *bla*_TEM_, *bla*_SHV_, *bla*_OXA_, and *bla*_CTX−M−gp1_, *bla*_CTX−M−gp2_, and *bla*_CTX−M−gp9_, *bla*_TEM_ were the most common being present in majority of the MDR isolates and *bla*_OXA_, *bla*_CTX−M−gp1_, *bla*_SHV_, *bla*_CTX−M−gp9_, and *bla*_CTX−M−gp2_ in certain isolates. Additionally, prevalence of methicillin resistant *S. aureus* (MRSA), and vancomycin resistant *Enterococcus* species was also noted which is at par with the reports of other researchers (Mehrotra et al., 2000; Hartemann-Heurtier et al., 2004; Gadepalli et al., 2006; Janifer et al., 2013; Jia et al., 2014). TEM type β-lactamase has been reported in earlier studies which also support our findings (Jia et al., 2014; Farid et al., 2015).

Of 38 isolates showing resistance to more than eight antibiotics, 26 (68.42%) isolates were positive for class 1 integron. This suggests that integrons are widely distributed in bacteria infecting DFUs. This is supported from the results of PCR amplification of 11 variable regions of class 1 integron in different isolates. Two types of class 1 integrons of varying sizes (0.5–4.0 kb) were detected in 4 MDR bacteria and 22 isolates possessed one integron of varying sizes (0.3–2.5 kb). It was also noted that variable region of class 1 integron carried one to three resistance genes. Altogether, 12 different gene cassettes encoding resistance to aminoglycosides and trimethoprim were detected. Presence of these two gene cassettes in MDR bacteria isolated from different sources has been reported (Antunes et al., 2006). Interestingly, our results showed that aminoglycoside adenyltransferase gene (*aad*A), which confers resistance to streptomycin and/or spectinomycin, was the most common among all the gene cassettes. Widespread resistance to aminoglycoside *viz*. streptomycin and/or spectinomycin may be due to the fact that they are extensively used for treating various infectious diseases including urinary tract infection and DFUs. Lately the therapeutic uses of streptomycin and spectinomycin are avoided but they are widely used in agriculture and livestock. The *dhfr* gene cassettes that confer resistance to trimethoprim were also detected frequently. Similar to our findings high prevalence of the aminoglycoside resistance (*aad*A) and trimethoprim resistance determinants (*dhfr*A) have been reported in *E. coli, K. pneumoniae* and *S. aureus* from Asia and Europe (Sáenz et al., 2004; Gadepalli et al., 2006; Chang et al., 2009; El-Najjar et al., 2010). Additionally, clinical isolates namely *E. coli, S. haemolyticus, Enterococcus* spp., *Pseudomonas* spp., and *Alcaligenes* spp. have also been reported to carry resistance to trimethoprim, streptomycin and spectinomycin (White et al., 2001; Lindstedt et al., 2003; Nógrády et al., 2005). The *dhfr*AXVII- *aadA*V gene cassette was present in the variable region of 12 integron-positive isolates, where the location of *aadA*V was close to the 3′CS and *dhfr*AXVII to the 5′CS region. Strong association between the presence of gene cassettes and resistance to specific antibiotics have been demonstrated in several studies (Chang et al., 2000; Roe and Pillai, 2003; Rao et al., 2008). On the contrary, in our study certain isolates were found to carry integron gene cassettes but the corresponding antibiotic resistance phenotype was absent. This might be due to the inefficient expression of the inserted gene cassettes by the integron promoter. Result showing the occurrence of integrons with identical cassettes (*dhfrA*XVII, *aadA*V) in a number of isolates is an interesting finding of this study. It seems that certain species may have identical mechanism (s) for the acquisition of multi-resistance to antibiotics. To our knowledge, occurrence of class 1 and 2 integrons in clinical isolates of *Enterococcus* spp has been reported only recently (Xu et al., 2010; Yan et al., 2010) and therefore occurrence of integron in *Eneterococcus* spp. in this study is new addition and supports the findings of earlier researchers.

## Conclusion

In conclusion, the occurrence of multiple-antibiotic resistant bacteria seems widespread in DFUs. Findings of this study clearly indicate that resistance to antibiotics is mediated mainly due to the presence of class 1 integrons. Furthermore, certain β-lactamases are specifically induced upon growth of MDR strains with antibiotics and may be important in conferring resistance to antibiotics. As the worldwide prevalence of antibiotic-resistant bacteria is on increase and may cause serious threat to human health, further study of integrons and their associated gene cassettes is needed to understand the mechanisms of acquisition of MDR genes in clinical isolates. Findings of the present study may provide useful insights for the selection of potential antibiotics and management of DFUs in diabetic patients.

### Conflict of interest statement

The authors declare that the research was conducted in the absence of any commercial or financial relationships that could be construed as a potential conflict of interest.

## Footnotes

^1^Clinical Laboratory Standard Institute (2010). *Performance Standards for Antimicrobial Susceptibility Testing. CLSI Approved Standards M100-S20*. Wayne, PA: CLSI.
